# Mutation and Selection of Prions

**DOI:** 10.1371/journal.ppat.1002582

**Published:** 2012-03-29

**Authors:** Charles Weissmann

**Affiliations:** Department of Infectology, Scripps Florida, Jupiter, Florida, United States of America; Washington University School of Medicine, United States of America

## Prion Diseases

Prion diseases, or transmissible spongiform encephalopathies (TSEs), occur naturally in several species, including humans, cattle, sheep, and deer, and can be transmitted experimentally to many others. Typically, incubation times are relatively long, extending to 40 years or more in humans; however, after appearance of clinical symptoms, death mostly ensues within less than a year, as a consequence of neurodegeneration accompanied by accumulation of abnormal conformers of the host protein PrP. Natural transmission usually occurs perorally, as exemplified by the kuru epidemic among the Fore people of Papua New Guinea, attributed to cannibalistic practices; the bovine spongiform encephalopathy (BSE) epizootic in the United Kingdom at the end of last century, caused by feeding of contaminated meat-and-bone meal to cattle; or the current epizootic of chronic wasting disease afflicting cervids in 19 states of the United States. Transmission of BSE prions to young humans gave rise to a limited outbreak of a novel illness, variant Creutzfeldt-Jakob disease (vCJD), almost exclusively in the UK. Sporadic cases of prion disease occur at very low frequency in human populations (sCJD) and in cattle herds (atypical BSE), and are attributed to spontaneous generation of prions in the affected individuals. Finally, familial forms of human prion disease are linked to a variety of different, dominant mutations in the *PRNP* gene, and while afflicted families are rare, penetrance is very high.

## Replication of Prions

**Figure 1:**
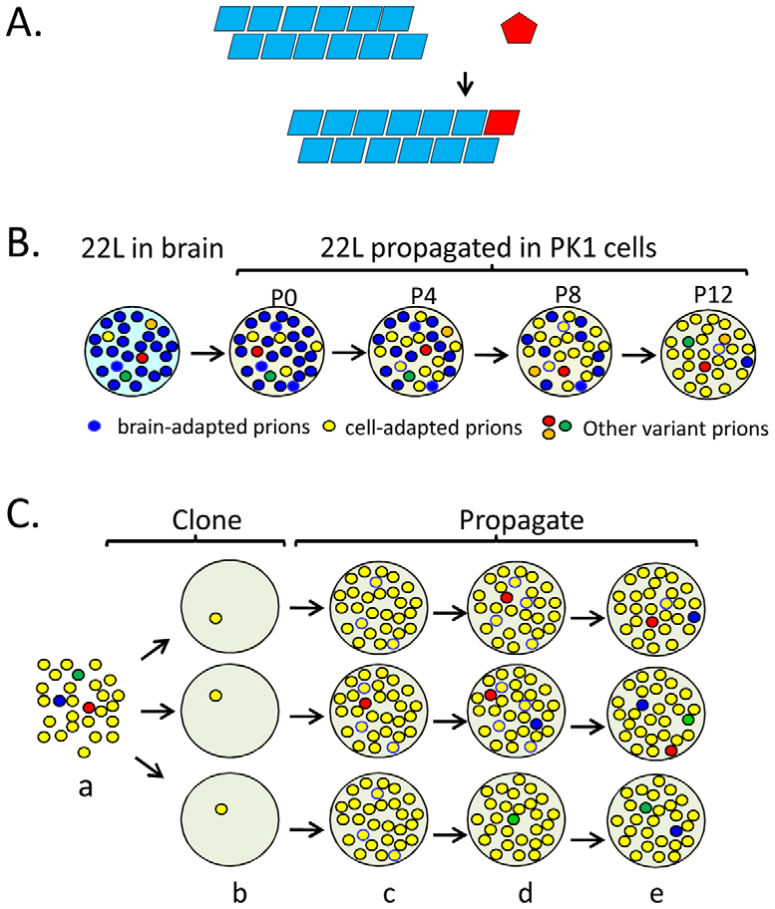
Propagation, mutation, and selection of prions in cultured cells. (A) The seeding model of prion propagation predicates that PrP^C^ monomers add to the termini of PrP^Sc^ fibrils and in doing so, adopt the conformation of the constituent PrPSc subunits. (B) Prion populations are thought to constitute quasi-species, consisting of a major species and numerous variants at low levels. Brain-adapted 22L prions are resistant to swainsonine treatment when assayed on PK1 cells and are able to infect R33 cells (R33 competent). When propagated in PK1 cells, swainsonine-sensitive, R33-incompetent prions gradually (passages P0 to P12) become the major species in the population because they multiply faster. (C) PK1 cell-adapted 22L prions (a) were cloned (b) in PK1 cells. The populations become heterogeneous as mutations arise during propagation (c–e). The red circles represent swainsonine-resistant prions; when challenged with the drug, some populations (top and middle row) acquire the capacity to become resistant while others (bottom row) do not. Schematic representation of data from reference [16].

Prions consist mainly, if not solely, of PrP^Sc^ (scrapie prion protein), aggregated conformers of the GPI-linked host glycoprotein PrP^C^ (cellular prion protein). PrP^Sc^ propagates by converting PrP^C^ to a replica of itself (Figure 1A). PrP^C^ may exist as an equilibrium mixture of conformers, some of which can accrete to PrP^Sc^ “seeds” at a critical rate [1], [2]. This seeding model is supported by the protein misfolding cyclic amplification (PMCA) reaction, in which brain homogenate, as a source of PrP^C^, is spiked with a seed of infected brain homogenate and subjected to multiple cycles of sonication and incubation, ultimately yielding a vast excess of infectious prions [3]. Infectious prions arose spontaneously in PMCA-mediated, cell-free reactions from defined components [4], in particular from recombinant PrP, a phospholipid, and poly(A) or poly(dT) [5], definitively laying to rest the perennial proposal that the infectious agent is a virus-like entity [6]. Prion-like, seeded conversion into an aggregated state has been proposed for several mammalian proteins such as Abeta, α-synuclein, or serum amyloid, which underlie protein misfolding diseases, and for several fungal, in particular yeast, proteins.

## Prion Strains

Prion populations may present as distinct strains: these differ in their phenotypic properties but are associated with PrP^Sc^ having the same amino acid sequence. Murine prion strains, originally characterized by the incubation time and the neuropathology they elicit, can be propagated indefinitely in mice homozygous for the PrP gene. Many “classical” strains currently propagated in mice and hamsters, such as 79A, 22L, and ME7, originated from scrapie-infected sheep or goats [7] and were cloned by endpoint dilution in mice.

Strain-specific properties of the prion are believed to be enciphered in the conformation of the cognate PrP^Sc^ [8], and indeed, distinct strains are often associated with PrP^Sc^ species differing in physicochemical properties. Experiments with yeast prion strains have shown that specific conformations can be propagated in vitro by pure, unglycosylated proteins [9]. Nonetheless, in view of the vast multiplicity of mammalian prion strains and their tropism for particular cell lines, it is conceivable that post translational modifications of PrP, such as glycosylation or association with some cellular components, might favor certain PrP conformations and hence account for cell-specific preferential propagation of particular strains.

## The Species Barrier

In general, there is a considerable barrier to transmission of prions between animal species, in that even massive intracerebral trans-species inoculation causes disease at only low frequency (low “attack rate”) and/or only after very long incubation times, if at all. This barrier was abolished in some instances by replacing the PrP gene of the recipient by its counterpart from the donor, but clearly factors other than mismatch of PrP sequences contribute to the incompatibility. Importantly, when prions are serially transmitted from the initial trans-species recipients to further animals of the same species, attack rates increase and incubation times decrease, reflecting “adaptation” to the new host [10]. “Adaptation” implies as a first step accretion of PrP^C^ from the recipient host to the incoming PrP^Sc^ seed, which may be a very inefficient process if the amino acid sequence of the host PrP entrains a spectrum of conformations that are poorly compatible with that of the seed. Efficient propagation may only be enabled when the conformation of the seed changes, perhaps initially at the “growing end” [11], resulting in a “mutation” at the conformational level. Subsequently, prions may evolve to replicate more rapidly in the new host, accounting for the striking reduction of their incubation period as they are sequentially transferred within the new species.

In some instances, transfer of a prion strain from one species to another, followed by several passages in the original host species, led to emergence of mutant strains. For example, when cloned murine 139A prions were passaged through hamster and subsequently passaged repeatedly in mouse a new strain, 139A-H2M, was recovered; however, ME7 subjected to the same procedure remained apparently unchanged [12].

## Evolution of Prions

The finding that many murine prion strains replicated efficiently in selected murine cell lines created important new experimental opportunities. In particular, the slow, expensive, and imprecise mouse-based bioassay for murine prions could be replaced by a humane, rapid, and precise cell-based procedure, the standard scrapie cell assay (SSCA) [13]. The differential susceptibility of cell lines to various prion strains provided the basis of the cell panel assay (CPA), which rapidly differentiates between various prion strains on the basis of their cell tropism and their susceptibility to various drugs, such as swainsonine or kifunensine [14], [15].

**Figure 2:**
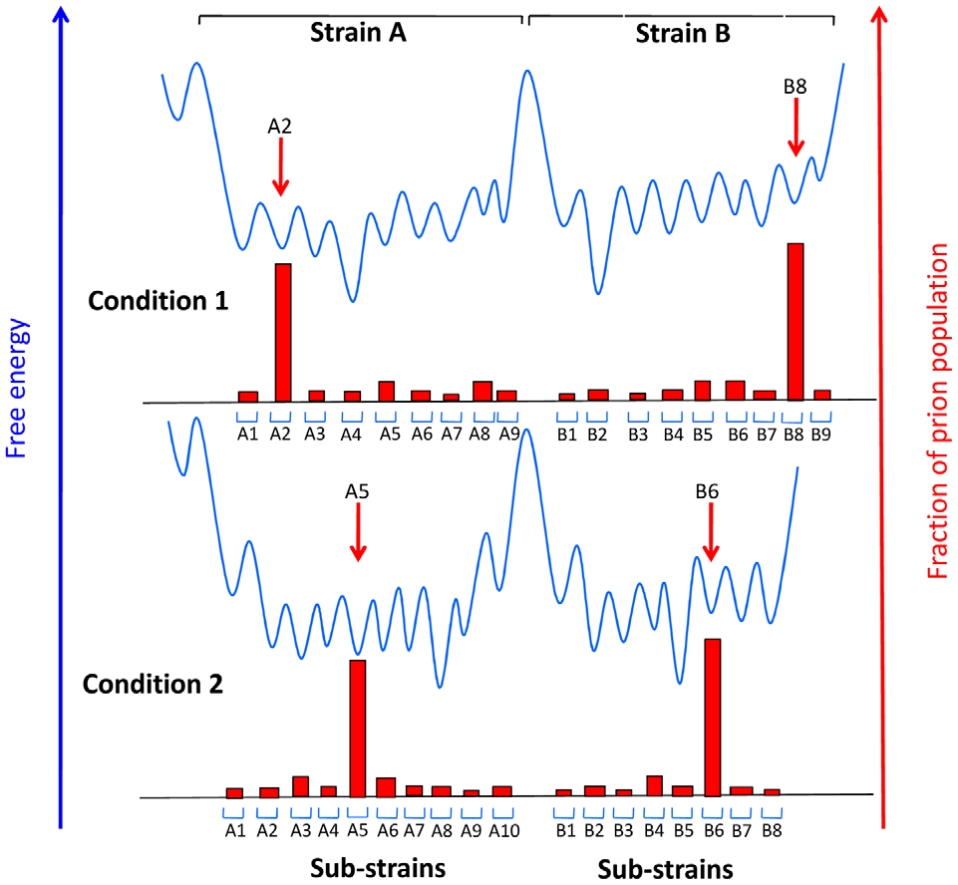
Conjectural free energy landscape for prion strains and sub-strains. Sub-strains are depicted as distinguishable collectives of prions that can interconvert readily because they are separated by activation energy barriers that can be overcome in a particular environment under physiological conditions, while strains are separated by high energy barriers. The extent to which the individual wells are populated (red blocks) is determined by the accumulation rate of the particular sub-strain. When the environment changes, for example when prions are transferred between distinct tissues, different sub-strains may be favored. Adapted from reference [18].

The CPA revealed that serial propagation of brain-derived 22L prions in PK1 cells led to progressive change in their properties; while initially able to propagate in R33 cells (“R33 competent”) or in PK1 cells in the presence of swainsonine (“swainsonine resistant”), the prions gradually became completely R33 incompetent and swainsonine-sensitive (Figure 1B). When these “cell-adapted” prions were returned to mouse brain, they gradually re-acquired their former properties and became indistinguishable from the original 22L strain [16]. Along similar lines, when swainsonine-sensitive prions were propagated in PK1 cells in the presence of the drug, a swainsonine-resistant prion population emerged after a few passages, documenting adaptation to the new environment. After withdrawal of the drug, further propagation for several splits again yielded drug-sensitive prions [16]. These findings suggested that prion populations constitute so-called quasispecies [17], that is, they are composed of a variety of conformational variants, each present at a low level; when the environment changes, the most efficiently replicating variant becomes the predominant component of the population, which then constitutes a distinct sub-strain [1], [16], [18]. Indeed, PK1 cell-adapted 22L populations were found to contain about 0.5% swainsonine-resistant variants before ever being exposed to the drug [16]. Because the 22L prions used in these experiments had been cloned by endpoint dilution years earlier, heterogeneity must have arisen by a mutation-like process in the interim. Mutations in the case of prions represent conformational changes and not modifications at the level of the protein sequence, because PrP is encoded by the host genome and the mutation is inherent to the proteinaceous particle. To verify whether heterogeneity of prion populations came about by mutation, swainsonine-sensitive prions were cloned by endpoint dilution into PK1 cells, and the infected cells were propagated serially for up to 100 doublings and challenged with swainsonine to determine at which stage the prion populations acquired the capacity for becoming resistant to the drug. Early after cloning the populations were incapable of doing so, but most clones developed this capability after 31–86 doublings (Figure 1C). However, at least one of nine populations failed to do so even after 116 doublings, suggesting that the prions were heterogeneous in regard to their ability to develop swainsonine resistance [11], [16]. Acquisition of drug resistance by murine prions has also been reported by Ghaemmaghami et al. [19] and by yeast prions by Shorter [20]. Most if not all of the prion variants, or sub-strains, described above were reversible, suggesting that the underlying conformations were readily interconvertible. In contrast, strains are very stable, at least as long as they are propagated in the same species. As shown in Figure 2, this suggests a low activation energy barrier between sub-strains, readily surmountable under physiological conditions, while high activation energy barriers prevent conversion between strains.

## Concluding Thoughts

The finding that prions can acquire resistance to drugs has significant implications for drug design. Drugs targeted to PrP^Sc^ may have to be administered in combination, as in the case of viruses, in particular HIV. Alternatively, drugs could be targeted to bind and stabilize PrP^C^ or, in view of the finding that ablation of PrP^C^, at least in animals, is not detrimental to health [21], [22], to suppress its synthesis. At present no therapeutically useful drugs are available, but deepening insight into the molecular biology of prions may pave the way to novel approaches.
